# Characterization of SMAD3 Gene Variants for Possible Roles in Ventricular Septal Defects and Other Congenital Heart Diseases

**DOI:** 10.1371/journal.pone.0131542

**Published:** 2015-06-25

**Authors:** Fei-Feng Li, Jing Zhou, Dan-Dan Zhao, Peng Yan, Xia Li, Ying Han, Xian-Shu Li, Gui-Yu Wang, Kai-Jiang Yu, Shu-Lin Liu

**Affiliations:** 1 Genomics Research Center (one of the State-Province Key Laboratory of Biopharmaceutical Engineering, China), Harbin Medical University, Harbin, China; 2 Intensive Care Unit, The Second Affiliated Hospital of Harbin Medical University, Harbin, China; 3 Department of Colorectal Surgery, the Second Affiliated Hospital of Harbin Medical University, Harbin, China; 4 Daqing People’s Hospital, Daqing, China; 5 Department of Microbiology, Immunology and Infectious Diseases, University of Calgary, Calgary, Canada; New York Medical College, UNITED STATES

## Abstract

**Background:**

Nodal/TGF signaling pathway has an important effect at early stages of differentiation of human embryonic stem cells in directing them to develop into different embryonic lineages. SMAD3 is a key intracellular messenger regulating factor in the Nodal/TGF signaling pathway, playing important roles in embryonic and, particularly, cardiovascular system development. The aim of this work was to find evidence on whether *SMAD3* variations might be associated with ventricular septal defects (VSD) or other congenital heart diseases (CHD).

**Methods:**

We sequenced the *SMAD3* gene for 372 Chinese Han CHD patients including 176 VSD patients and evaluated SNP rs2289263, which is located before the 5’UTR sequence of the gene. The statistical analyses were conducted using Chi-Square Tests as implemented in SPSS (version 13.0). The Hardy-Weinberg equilibrium test of the population was carried out using the online software OEGE.

**Results:**

Three heterozygous variants in *SMAD3 *gene, rs2289263, rs35874463 and rs17228212, were identified. Statistical analyses showed that the rs2289263 variant located before the 5’UTR sequence of *SMAD3* gene was associated with the risk of VSD (P value=0.013 <0.05).

**Conclusions:**

The SNP rs2289263 in the *SMAD3* gene is associated with VSD in Chinese Han populations.

## Introduction

Congenital heart diseases (CHD) include a series of congenital anatomic malformations, such as pulmonary stenosis, tetralogy of Fallot, patent ductus arteriosus, mitral valve insufficiency, atrial septal and ventricular septal defects etc. [1], which may be complicated by arrhythmias or heart failure and increase the risks of coronary heart diseases [2]. The clinical characteristics of CHDs are very complex, and the diseases have high morbidity and mortality. The incidence of all types of CHDs is about 7.5 present of newborns [3], many of which require clinical intervention (about 1%) [4]. Of all CHD patients, only about 13% are reported with chromosomal variants [5], so currently surgery is still the main treatment for CHD [6]. Although many genetic defects have been revealed in many familiar and sporadic CHD cases by extensive genetic studies [7, 8], the relationships between genetic abnormalities and CHD etiology remain largely unknown.

Ventricular septal defects (VSDs) account for about 40% of CHDs [1, 4]. The prevalence of VSDs varies in different studies due presumably to differences in diagnostic methods and age of participants [9–11]. VSDs may also be associated with other structural cardiac defects or syndromes, such as aortic coarctation or interruption, tetralogy of Fallot, univentricular atrioventricular connection and Down syndrome [12, 13]. Recently the prevalence of VSDs is increased in newborns due to changes in diagnosis and screening modalities such the use of fetal echocardiography [14, 15].

The heart is among the first formed organs during the embryogenesis and its formation is strictly controlled by gene regulatory networks consisting of many signaling pathways, transcription factors, epigenetic factors, and miRNAs [16, 17]. A large number of defects in genes coding for these factors have been identified [1, 18]. Recently, we reported that SNP rs2295418 in the *Lefty2* gene and genotype frequency of rs360057 in *Lefty1* gene are associated with the risk of CHD [1]. LEFTY is a crucial transforming growth negative regulation factor in the Nodal/TGF-βsignaling pathway [19], which inhibits the cellular proliferation and differentiation [20, 21]. Importantly, the Nodal/TGF-βsignaling pathway has an important effect in early stages of differentiation of the human embryonic stem (HES) cells, directing them to develop into different embryonic lineages, and errors in the transformation may occur if the pathway has malfunctions [22–24].

The HES cells differentiate to various cell types, which develop to ectoderm, endoderm and mesoderm; generation and differentiation of cardiomyocytes and muscle cells take place in the mesoderm [25]. As a key intracellular regulating factor in the Nodal/TGF-βsignaling pathway, SMAD family member 3 (SMAD3) activates or represses gene transcription, thus having important effects on embryonic development that will influence the formation of the cardiovascular system [26, 27]. At the same time, some authors also suggest that Nodal/TGF-βsignaling pathway plays a key roles in the embryogenesis of the heart, valvular pathogenesis and organization of the aortic wall; when activities of the signaling pathway were disrupted, CHDs ensued in animal studies [28]. For elucidation of the mechanisms, the *SMAD3* gene knockdown mice could be used as models [29].

To validate possible associations of *Smad3* with VSD or other CHDs, we analyzed the transcribed region and splicing sites of the gene and compared the gene sequences between 372 Chinese Han CHD patients (including 176 VSD patients) and 456 controls. We found that the rs2289263 variant before the 5’UTR of the *Smad3* gene was closely associated with the risk of VSD but not with the other CHDs.

## Results

### Patients

We confirmed the clinical diagnosis of all the recruited patients in Linyi people’s Hospital, the second Affiliated Hospital and the fourth Affiliated Hospital of Harbin Medical University. The CHD patients had no history or manifestations of any other systemic abnormalities. We established that their mothers did not have a history of taking medicines or attracting infections during gestation, as those factors have been shown to be associated with heart malformation in pregnancy [30, 31].

The 372 CHD patients contained 176 with ventricular septal defects (VSD), 14 with tetralogy of Fallot, 12 with pulmonary stenosis, 25 with patent ductus arteriosus, 22 with mitral valve insufficiency, 53 with atrial septal defects, and 70 with other complex congenital heart defects. All the CHD patients (n = 372, male 201, female 171, the min and max age were 0.2 and 74 respectively, and the average age was 15.22 years) and unrelated controls (n = 456, male 257, female 199, the min and max age were 0.25 and 41 respectively, and the average age was 14.66 years) were recruited for this study, and there were no statistical differences of the gender composition or age between the two groups (Table 1).

**Table 1:** Clinical characteristics of study population.

*Parameter*	*CHD*	*Control*	*F*	*t*	*P*	*95%CI Up*	*95%CI Low*
***Sample (n)***	372	456	None	None	None	None	None
***Male/Female (n)***	201/171	257/199	None	None	0.527	None	None
**Age (years)**	15.22±15.24	14.66±10.07	90.776	0.654	0.513	-1.14543	2.28965

Data are shown as mean±SD; between the two groups, there were no statistical differences of the age and gender composition.

### *SMAD3* gene analysis

**Fig 1:**
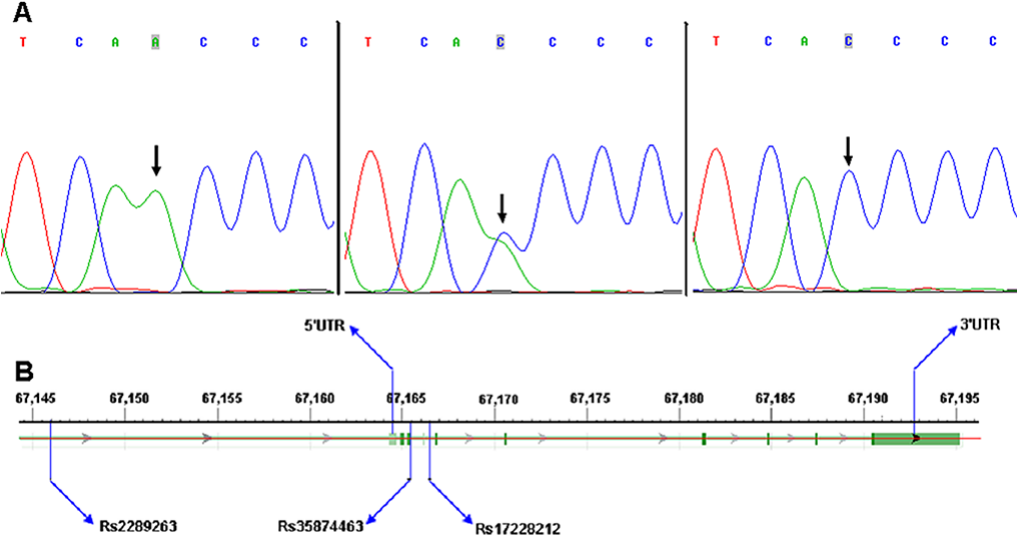
Schematic diagrams and DNA sequence chromatograms. A: Schematic diagrams of rs2289263, rs35874463 and rs17228212 locations in the SMAD3 gene; B: Three genotypes of DNA sequence chromatograms of rs2289263.

We sequenced the *SMAD3* gene to test the hypothesis that germline common genetic variants in *SMAD3* may confer susceptibility to CHD. We first compared the transcribed region and splicing sites of *SMAD3* and found the variation rs35874463 was located within the translated region and rs17228212 was located within an intron of the SMAD3 gene, but the genetic heterozygosity of the two SNP were very low (S1 Table). The variation rs2289263 was located before 5’UTR of the gene, but its genetic heterozygosity was very high (Fig 1A).

### SNP rs2289263 genotyping statistical analysis

To further test any possible associations between *SMAD3* and CHD, we conducted SNP analyses and found that the variant rs2289263 before 5’UTR of *SMAD3* gene was associated with the risk of VSD in the Chinese Han population but not with the other CHDs (Tables 2 and 3). At the same time, Hardy-Weinberg equilibrium test for the CHD and controls were conducted and it was in line with the equilibrium.

**Table 2:** The genotype and allele frequency of SNP rs2289263 in 372 CHD patients, 176 VSD patients and 456 non-CHD controls.

*Group*		*Genotype frequency (%)*			*Allele frequency (%)*	
Genotype		A/A	A/C	C/C	A	C
CHD	372	136(36.6)	168(45.2)	68(18.3)	440(59.1)	304(40.9)
Controls	456	141(30.9)	228(50.0)	87(19.1)	510(55.9)	204(44.1)
VSD	1760	72(40.9)	80(45.5)	24(13.6)	224(63.6)	128(36.4)

**Table 3:** SNP rs2289263 before 5’UTR of *SMAD3* gene associated with the risk of ventricular septal defect not congenital heart diseases in Chinese populations.

*Title*		*Pearson Chi-square*				*Spearman Correlation*			
Comparison Group	Type	Value	Min count^a^	df	Asymp. Sig. (2-sided)	Value	Asymp. Std. error^b^	Approx. T^c^	Approx. Sig
***CHD- Controls***	Genotype	3.020^a^	69.64	2	0.221	-0.045	0.035	-1.303	0.193^d^
	Allele	1.736^a^	317.19	1	0.188	-0.032	0.025	-1.317	0.188^d^
***VSD- Controls***	Genotype	6.493^a^	30.91	2	0.040	-0.100	0.039	-2.530	0.012^d^
	Allele	6.209^a^	147.59	1	0.013	-0.070	0.028	-2.496	0.013^d^

a: The minimum expected count

b: Not assuming the null hypothesis

c: Using the asymptotic standard error assuming the null hypothesis

d: Based on normal approximation.

## Discussion

In this study, we analyzed the transcribed regions and splicing sites of the *SMAD3* gene in a large cohort of CHD patients and controls and found that the variant rs2289263 in the *SMAD3* gene was associated with the risk of VSD in the Chinese Han population, demonstrating the involvement of the *SMAD3* gene in the VSD etiology.

Eighteen or nineteen days after fertilization, the human heart starts to form in the mesoderm, and the formation involves strict temporal, spatial, and sequential expression of genes [1]. Nodal/TGF-βsignaling pathway plays a key role during the mammal gastrulation to produce progenitor cells of the mesendoderm [32]. In this process, the expression level of Nodal/TGF-βsignaling pathway can affect the formation of mesendoderm [33].

The mesendoderm progenitor cells form the primitive streak and mutations in the *Nodal* gene can affect the formation. In mice, the vascular systems arise from extraembryonic mesoderm and migrate through the primitive streak to the presumptive yolk sac [34]. The *Nodal* gene expression initiates a series of signal transduction and induces some gene and its own expression in later stages of embryonic development [19, 32]. Animal studies also show that TGF-β can induce cardiac fibroblasts proliferation, myocardial fibrosis and cardiomyocytes hypertrophic growth [35], and loss of responsiveness to TGF-βmay lead to fibrosis progresses in the atrial fibrogenesis [14, 36]. Furthermore, many lines of evidence from animal research suggest that TGF-βsignaling is essential in the cardiogenesis, valvular pathogenesis, and organization of the aortic wall [28, 37], and disrupted TGF-βsignaling activities may lead to congenital heart defects [28].

We analyzed genes of the Nodal/TGF-βsignaling pathway, as they have been demonstrated to play vital roles in mesoderm differentiation and heart formation [32]. In this work, we found that the variant rs2289263 before the 5’UTR of *SMAD3* gene was associated with increased risk of VSD in the Chinese Han population, while in a previous study, we demonstrated that rs2295418 (g.C925A) in *Lefty2* gene is associated with the risk of CHD [1]. Of great significance, LEFTY and SMAD3 both play central roles in the Nodal/TGF-βsignaling pathway, with LEFTY negatively regulating the Nodal/TGF signaling pathway and *SMAD3* defects being associated with cardiovascular diseases [26, 29]. To our knowledge, this is the first report showing the association of *SMAD3* gene with VSD or other congenital heart defects. Further work will be needed on the the Nodal/TGF-βsignaling pathway genes such as *LEFTY* and *SMAD3* for their involvement in the pathogenesis of CHD at the molecular level.

## Materials and Methods

### The study population

From Linyi People’s Hospital, the second Affiliated Hospital and the fourth Affiliated Hospital of Harbin Medical University, Harbin, China, we collected specimens of 372 CHD patients including 176 with VSDs for this study. The 456 controls with no reported cardiac phenotypes were also recruited for this study from the Medical Examination Center of the Second Affiliated Hospital of Harbin Medical University (Table 1). All the CHD subjects and controls received comprehensive physical examination, electrocardiogram and ultrasonic echocardiogram examinations. None of the patients showed any other abnormalities in the heart or other body parts and the control members did not have any defects in the heart. From each participant or their parents on behalf of minors, we obtained a written informed consent, and the Ethics Committee of the Harbin Medical University approved this work, consistent with the 1975 Declaration of Helsinki.

### DNA analysis

We used standard protocols to extract genomic DNA from the peripheral blood leukocytes of the participants. The human *SMAD3* gene consisting of nine exons is located on 15q21-22. Using two stage methods, we determined the SNP genotypes in the *SMAD3* gene. First, the nine exons and the splicing sites of the gene were amplified using polymerase chain reaction (PCR) method (Table 4), and the products were sequenced using standard protocols [38]. After that, the genotypes of the SNP were determined using PCR and gene sequencing methods [1].

**Table 4:** PCR primers used for *SMAD3* gene sequence analysis.

*Exon*	*Forward primer*	*Reverse primer*	*Size*	*Tm*
1	GCGAAGTTTGGGCGACCG	GTGCCGCGTGGAAGCCTC	553	52.3
2	ATGGCCGGTTGCAGGTGT	CAGAGGTGGCTCAGTGTCG	331	57.6
3	GACTTTGGTGCTGGTCTGG	GGGAGCTGAGGTCATGGGT	383	57.8
4	AGAGCCAAGCTGTGAAGG	AGAGGAAGGGATGGAAGG	203	52.8
5	TGGGCTACCCCTCCTTGA	GGCTGAGCTGGGCTGATG	271	56.0
6	GAGGGAGCATGGGGCTTGG	GGGGTGGGATAGAGTGGC	329	57.6
7	TTAGGCTTGGGCTTTGGG	GGTTAAAGGCAGACCTATCAG	512	55.5
8	AGGAGATGGGTTCAAGGG	TGCCAGCAAACATCGTTC	563	55.9
9	GTTTGGCCGGGTAGTTTC	ACCTCTGGGTTTGCTCGT	462	53.7

### Rs2289263 SMAD3 SNP genotyping analysis and statistical methods

Using two stage methods, we determined genotypes of the rs2289263, rs35874463, and rs17228212 SNP of the *SMAD3* gene (Fig 1B). All the measurements were conducted by two independent researchers (Table 5). And then overall CHD meta-analysis was conducted and stratified analysis was carried out according to the types of CHD and sample sizes.

**Table 5:** PCR primers used for SNP statistical analysis.

*SNP*	*Forward primer*	*Reverse primer*	*Size*	*Tm (℃)*
***rs2289263***	CAACTCTGCCTGGCTGTA	CTCCATTTCTCCCTCCTG	132bp	52.1
***rs35874463***	GGGACTTTGGTGCTGGTCT	TCACGCTGCTCCTCTATGC	428bp	57.9
***rs17228212***	TAATCCTGCTGCGTTCCT	CCCTTTGGTCCCTACTATCT	372bp	52.9

The continuous variable (measurement data, such as age) statistical analyses were conducted using independent-samples T test and the discrete variable (enumeration data, such as gender composition and genotype frequency) statistical analyses were conducted using Chi-Square Tests to calculate odds ratios and P value as implemented in SPSS (version 19.0). P values less than 0.05 were considered statistically significant. The Hardy-Weinberg equilibrium test of the CHD and control population was conducted with the online software OEGE.

## Supporting Information

S1 Table The genotype and allele frequency of SNP rs35874463 and rs17228212 in 372 CHD patients and 456 non-CHD controls. (DOC)
