# Supplementary material for: Characterization of SMAD3 Gene Variants for Possible Roles in Ventricular Septal Defects and Other Congenital Heart Diseases
Source: PLoS One. 2015 Jun 25;10(6):e0131542. doi: 10.1371/journal.pone.0131542 (PMC4482402; doi:10.1371/journal.pone.0131542)
Supplement: S1 Table — (DOC) [file pone.0131542.s001.doc]

Supporting Information**:**

**S1 Table：**The genotype and allele frequency of SNP rs35874463 and in 372 CHD patients and 456 non-CHD controls

| ***Group*** | | ***Genotype frequency (%)*** | | | ***Allele frequency (%)*** | |
| --- | --- | --- | --- | --- | --- | --- |
| rs35874463 Genotype | | A/A | A/G | G/G | A | G |
| Group | No. |
| CHD | 372 | 370(99.5) | 2(0.5) | 0 | 742(99.7) | 2(0.3) |
| Controls | 456 | 454(99.6) | 2(0.4) | 0 | 910(99.8) | 2(0.2) |
| rs17228212 Genotype | | T/T | T/C | C/C | T | C |
| Group | No. |
| CHD | 372 | 366(98.4) | 6(1.6) | 0(0) | 738(99.2) | 6(0.8) |
| Controls | 456 | 451(98.9) | 5(1.1) | 0(0) | 907(99.5) | 5(0.5) |
